# Safety and Tolerability of Vildagliptin 100 mg Sustained-Release Formulation in Type 2 Diabetes Mellitus: Results From a Prospective, Multicenter, Single-Arm, Post-marketing Surveillance Study

**DOI:** 10.7759/cureus.107564

**Published:** 2026-04-23

**Authors:** Pravin Soni, Ashutosh Sonawane, Sandeep Gaidhani, Niddhi Baxi, Deeksha Malhotra, Shivani Acharya

**Affiliations:** 1 Medicine, Yashwantrao Chavan Memorial Hospital, Pune, IND; 2 Internal Medicine, Sarthak Health Clinic, Nashik, IND; 3 Critical Care Medicine, Life Care Hospital, Nashik, IND; 4 Medical Affairs, Abbott Healthcare Pvt. Ltd., Mumbai, IND; 5 Clinical Development and Operations, Abbott Healthcare Pvt. Ltd., Mumbai, IND

**Keywords:** adherence, dpp-4, effectiveness, sustained release, type 2 diabetes, vildagliptin

## Abstract

Background: Management of type 2 diabetes mellitus (T2DM) often requires multiple medications and complex dosing schedules, which can compromise adherence, especially in those with multiple comorbidities. Strategies that reduce pill burden and dosing frequency may enhance patient convenience and compliance. Vildagliptin administered as a 100 mg sustained-release (SR) formulation once daily offers an alternative to the conventional 50 mg twice-daily regimen. This prospective post-marketing surveillance study evaluated the safety and tolerability of vildagliptin 100 mg SR over 24 weeks in patients with T2DM.

Methods: This was a prospective, multicenter, single-arm, open-label study. Adults (≥18 years) with a clinical diagnosis of T2DM were prescribed vildagliptin 100 mg sustained-release once daily. The incidence of adverse drug reactions (ADRs) and mean changes in glycated hemoglobin (HbA1c), fasting plasma glucose (FPG), postprandial glucose (PPG), alanine aminotransferase (ALT), aspartate aminotransferase (AST), serum creatinine, serum amylase, and serum lipase were evaluated at 12 and 24 weeks.

Results: A total of 210 patients (129 males and 81 females) with a mean (SD) age of 52.63 (10.77) years were enrolled. A total of nine ADRs were reported in seven patients (3.3%), corresponding to an overall incidence of 4.3%. All events were mild in intensity, non-serious, and assessed as having a probable or likely relationship with the study drug. The mean HbA1c level decreased significantly from 7.89 at baseline to 7.27 at week 12 and 7.06 at week 24 (both p<0.001). Mean FPG levels at baseline, week 12, and week 24 were 145.92, 138.29, and 135.82 mg/dL, respectively, while the corresponding PPG levels were 201.84, 186.62, and 180.99 mg/dL, respectively. The p-values for the declines in both parameters at both time points were <0.001. There were no clinically meaningful changes (p>0.05) in AST and serum creatinine levels. The mean serum alanine aminotransferase (ALT) level decreased from 29.08 U/L to 27.76 U/L (p<0.05) at week 24. Similarly, mean serum amylase and lipase levels decreased from 59.36 U/L and 49.06 U/L to 56.50 U/L (p<0.05) and 45.38 U/L (p<0.01), respectively, at week 24.

Conclusion: This study demonstrated that vildagliptin 100 mg SR once daily monotherapy given over 24 weeks was well tolerated and effective in patients with T2DM. It effectively controlled glycemic parameters with no significant adverse events or harmful renal or hepatic effects.

## Introduction

Dipeptidyl peptidase-4 (DPP-4) regulates glucose metabolism by degrading incretin hormones, including glucagon-like peptide-1 (GLP-1) and gastric inhibitory polypeptide (GIP), which are secreted in response to nutrient intake [1, 2]. GLP-1 triggers most of the post-meal insulin secretion. However, its half-life is only 2 minutes, and it is rapidly degraded by the DPP-4 enzyme [3]. The "gliptin" or DPP-4 inhibitor (DPP4i) class of drugs prevents degradation of GLP-1 and increases its bioavailability, thus prolonging insulin secretion [3]. Fifty percent inhibition of the DPP-4 enzyme extends the half-lives of GLP-1 and GIP by two times, 90% inhibition extends them by 10 times, and 95% inhibition increases them by 20 times [4]. In addition, DPP4i slows gastric emptying and reduces food intake [2]. Due to this unique mechanism of action, a low risk of hypoglycemia, and weight neutrality, DPP4is are widely used as monotherapy or in combination with metformin for controlling blood glucose in patients with type 2 diabetes mellitus (T2DM) [5]. DPP4is have been found to be more effective in Asians in general and Indians in particular [6-8], leading to their wide use in India.

Vildagliptin is one of the widely used DPP4is [9]. Based on its pharmacokinetic (PK) and pharmacodynamic (PD) properties, international and Indian guidelines recommend vildagliptin as a twice-daily dose [10, 11]. A dose of 50 mg vildagliptin inhibits ≥80% of DPP-4 for 12 hours [12, 13], while a single dose of 100 mg inhibits ≥80% of DPP-4 for about 15-16 hours [13]. Therefore, for maximal (≥80%) DPP-4 inhibition for 24 hours, vildagliptin is usually recommended as a 50 mg twice-daily dose [14]. However, patients with T2DM often require more than one antidiabetic agent to control their blood glucose and are often on polypharmacy due to various other comorbidities associated with T2DM [2, 5, 9]. Polypharmacy and multiple doses are associated with poor adherence, especially in patients with comorbidities [15, 16]. Reducing the dosage frequency to once daily (OD) can improve patient compliance with pharmacotherapy [2, 3, 9].

Vildagliptin given once daily as a 100 mg sustained-release (SR) formulation can reduce the pill burden [4]. The bioequivalence of vildagliptin 100 mg SR once daily with vildagliptin 50 mg twice daily has already been established in various studies [2, 3, 5, 9]. Previous studies have also compared the efficacy and safety of vildagliptin 100 mg SR OD with 50 mg BID in Indian patients [17-20]. However, except for the study by Chawla et al. [20], all other studies used vildagliptin as combination therapy [17-19]. Thus, there are inadequate data on the safety and efficacy outcomes of vildagliptin 100 mg SR OD used as monotherapy. The study by Chawla et al. [20], which retrospectively compared vildagliptin monotherapy at 50 mg twice daily with the 100 mg sustained-release (SR) once-daily formulation, was limited by potential confounding factors inherent to its design. Moreover, the study evaluated only short-term (12-week) outcomes and did not assess the renal or hepatic safety profile of the 100 mg SR dose. To address these gaps, we conducted a prospective post-marketing surveillance study to evaluate the safety and tolerability of vildagliptin 100 mg SR over 24 weeks in patients with T2DM.

## Materials and methods

Study design

This prospective, multicenter, single-arm, open-label, post-marketing surveillance study (CTRI/2022/10/046453; date of registration October 13, 2022) evaluated the safety and tolerability of 24 weeks of treatment with vildagliptin 100 mg SR once-daily formulation in Indian patients with T2DM. The study was conducted from July 2023 to June 2024 at seven sites across different geographical locations in India.

The study was conducted in compliance with the International Council for Harmonization guidelines on Good Clinical Practice (ICH-GCP) [21], applicable local regulatory standards, and the ethical principles of the Declaration of Helsinki. It also adhered to the New Drugs and Clinical Trials Rules, 2019 [22], established by the Ministry of Health and Family Welfare, Government of India, and relevant institutional standard operating procedures. Before study initiation, the protocol, informed consent documents, and any amendments were reviewed and approved by the appropriate ethics committee.

Patients

Eligible patients included adults (≥18 years) with a clinical diagnosis of T2DM who had been prescribed vildagliptin 100 mg sustained-release once-daily formulation by the treating physician as per approved labeling and routine clinical practice, and who provided written informed consent.

Patients were excluded if they had conditions in which vildagliptin use is contraindicated or not recommended, including type 1 diabetes mellitus, acute metabolic diabetic complications (e.g., ketoacidosis), New York Heart Association (NYHA) Class IV heart failure [23], hepatic impairment (including pre-treatment ALT or AST >2.5× the upper limit of normal), moderate-to-severe renal impairment or end-stage renal disease, or known hypersensitivity to vildagliptin or its excipients. These criteria were consistent with the approved prescribing information and standard clinical practice.

Study endpoints

The primary objective of this study was to evaluate the safety and tolerability of vildagliptin 100 mg SR once-daily formulation in Indian patients with T2DM. The primary safety endpoints were the incidence of adverse drug reactions (ADRs) and/or other pharmacovigilance-relevant information (OPRIs) during the 24-week treatment with vildagliptin 100 mg SR once-daily formulation.

ADRs were recorded based on spontaneous reporting during routine clinical practice. The severity of ADRs was assessed by the investigator as mild, moderate, or severe based on clinical judgment, and causality was evaluated using standard pharmacovigilance criteria, considering temporal association, alternative causes, and clinical plausibility. Clinically relevant laboratory abnormalities were reported as ADRs based on investigator assessment.

Effectiveness was the secondary objective of this study and was assessed through changes in key laboratory parameters. These included the mean change in glycated hemoglobin (HbA1c) (%), fasting plasma glucose (FPG), ALT, AST, serum creatinine, serum amylase, and serum lipase levels from baseline (Day 1) to week 24 (±2 weeks). The exploratory objective was to assess the mean change in postprandial glucose (PPG) levels over the same treatment period.

No proprietary clinical scales, scoring systems, or copyrighted assessment tools were used in this study; safety and effectiveness outcomes were evaluated using routine pharmacovigilance reporting and standard laboratory parameters.

Statistical analysis

Based on published data indicating a comparable incidence of adverse events with vildagliptin 100 mg once daily and placebo (30.4% and 34.4%, respectively) [24], the expected incidence of adverse events in this study was assumed to be 35% for vildagliptin 100 mg SR. A sample size of 207 patients provides a two-sided 95% confidence interval for a single proportion, calculated using the large-sample normal approximation, with a precision of ±6.5% around the expected proportion of 0.35.

All patients who received at least one dose of the study medication were included in the safety population. Patients in the safety population who completed at least one follow-up visit were included in the intention-to-treat (ITT) population. The per-protocol (PP) population consisted of patients in the ITT population who completed the study without major protocol deviations and was used for the effectiveness analysis.

Descriptive statistics (frequency (n) and percentages (%)) were used to summarize the safety parameters. Continuous variables under secondary objectives were summarized using the mean and standard deviation (SD). Changes in continuous variables from baseline to follow-up were analyzed using paired t-tests at a 5% level of significance (two-sided). All statistical analyses were performed using IBM SPSS Statistics for Windows, Version 29 (Released 2022; IBM Corp., Armonk, New York).

## Results

Demographics and baseline characteristics

**Figure 1:**
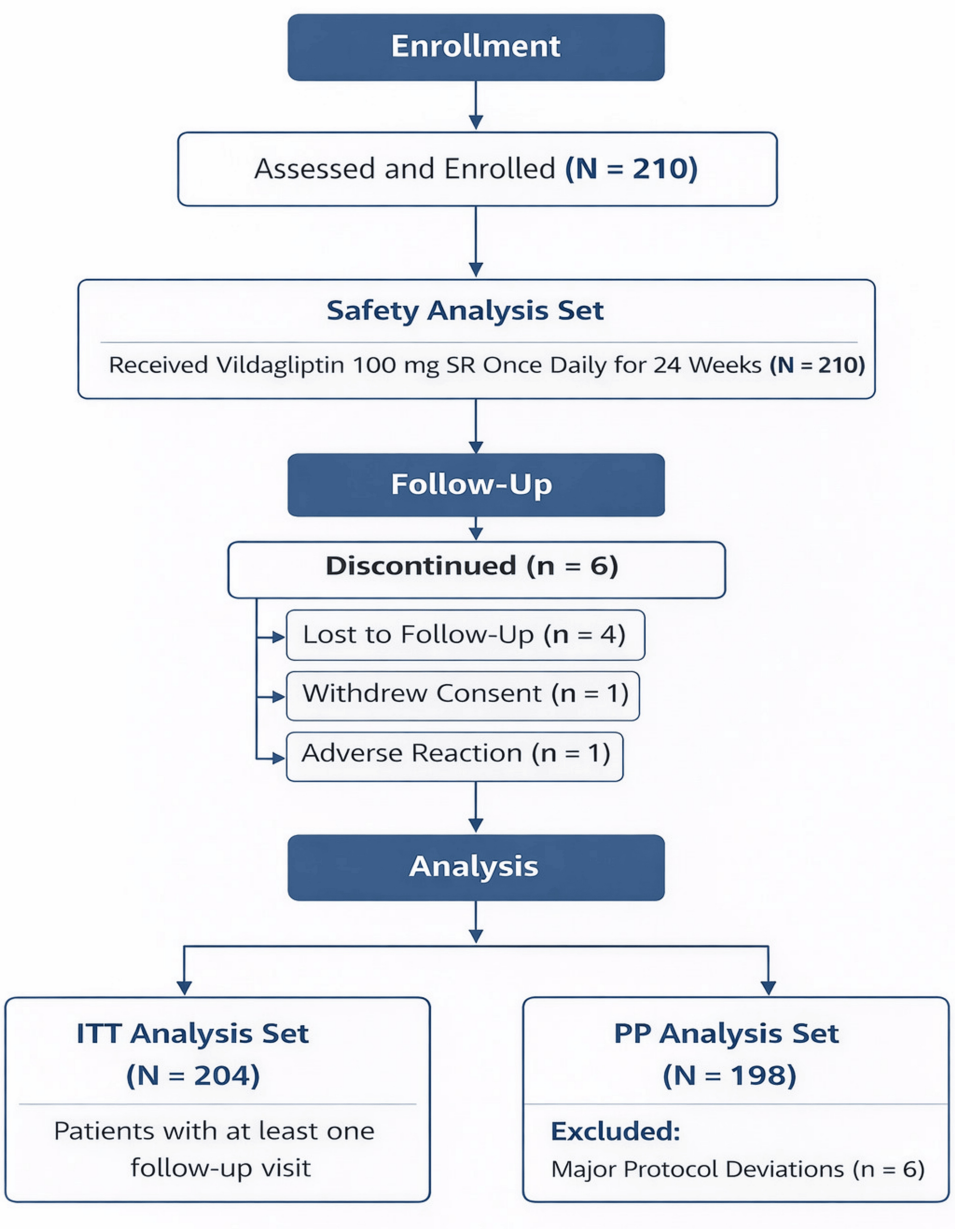
CONSORT flow diagram of study participants ITT, intention to treat; PP, per protocol.

A total of 210 patients (129 males and 81 females) with a mean (SD) age of 52.63 (10.77) years were enrolled in the study. All enrolled patients received vildagliptin 100 mg SR once daily for 24 weeks. Six patients dropped out of the study (four lost to follow-up, one withdrew consent, and one due to an ADR); hence, the intention-to-treat (ITT) set comprised 204 patients. Additionally, six patients with major protocol deviations were excluded from the secondary effectiveness analysis, yielding a per-protocol (PP) analysis set of 198 patients (Figure 1). 

The demographic and baseline characteristics of the subjects are summarized in Table 1.

**Table 1:** Patient demographics and baseline characteristics: safety population N is the number of patients in the Safety Set, and n is the number of patients with data available. Percentages are calculated using "N" as the denominator. BMI, body mass index; SD, standard deviation.

Parameter	Overall (N = 210)
Sex, n (%)	
Male	129 (61.4%)
Female	81 (38.6%)
Age (years), mean±SD	52.63±10.77
Height (m), mean±SD	1.63±0.08
Weight (kg), mean±SD	67.26±9.49
BMI (kg/m^2^), mean±SD	25.41±3.07

Primary outcome: safety

Safety was assessed on the basis of all ADRs, OPRIs, serious ADRs/OPRIs, and any events leading to discontinuation of treatment.

During the study, a total of nine ADRs were reported in seven patients (3.3%), corresponding to an overall incidence of 4.3%. The ADRs comprised elevated serum lipase levels (four events [1.9%]), elevated serum amylase levels (two events [1.0%]), and increased ALT, AST, and abnormal serum lipase levels (one event [0.5%] each). All events were mild in intensity, non-serious, and assessed as having a probable or likely relationship with the study drug. The study drug was discontinued in six patients (2.9%), two with elevated serum amylase and four with elevated serum lipase levels. One patient (0.5%) withdrew from the study due to an ADR. Of the nine ADRs, three (33.3%) resolved spontaneously during the study period, whereas the outcomes of the remaining six (66.7%) were unknown.

Secondary outcome

Significant improvement was observed across all glycemic parameters following 24 weeks of treatment with vildagliptin 100 mg SR once-daily formulation.

**Figure 2:**
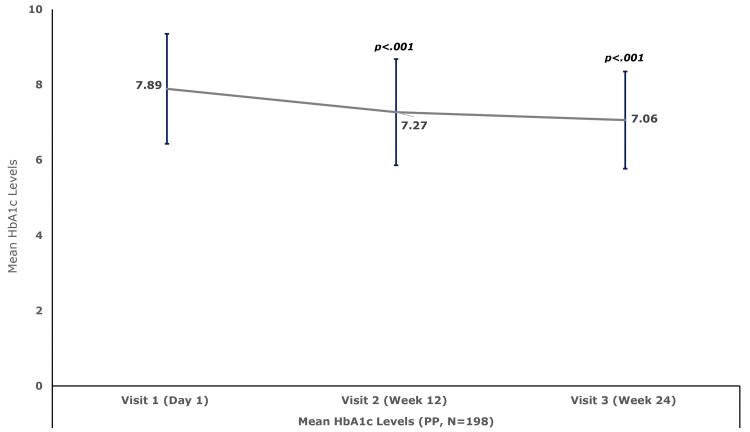
Mean (SD) hemoglobin A1c (HbA1c) levels (PP set, N = 198) N is the number of patients in the PP set. PP, per protocol; SD, standard deviation.

Compared to the baseline mean (SD) HbA1c level of 7.89 (1.48), the HbA1c level decreased significantly by 0.62 (0.95) (95% CI, −0.74 to −0.50; p<0.001) at week 12 and by 0.83 (1.07) (95% CI, −0.96 to −0.70; p<0.001) at week 24 (Figure 2).

**Figure 3:**
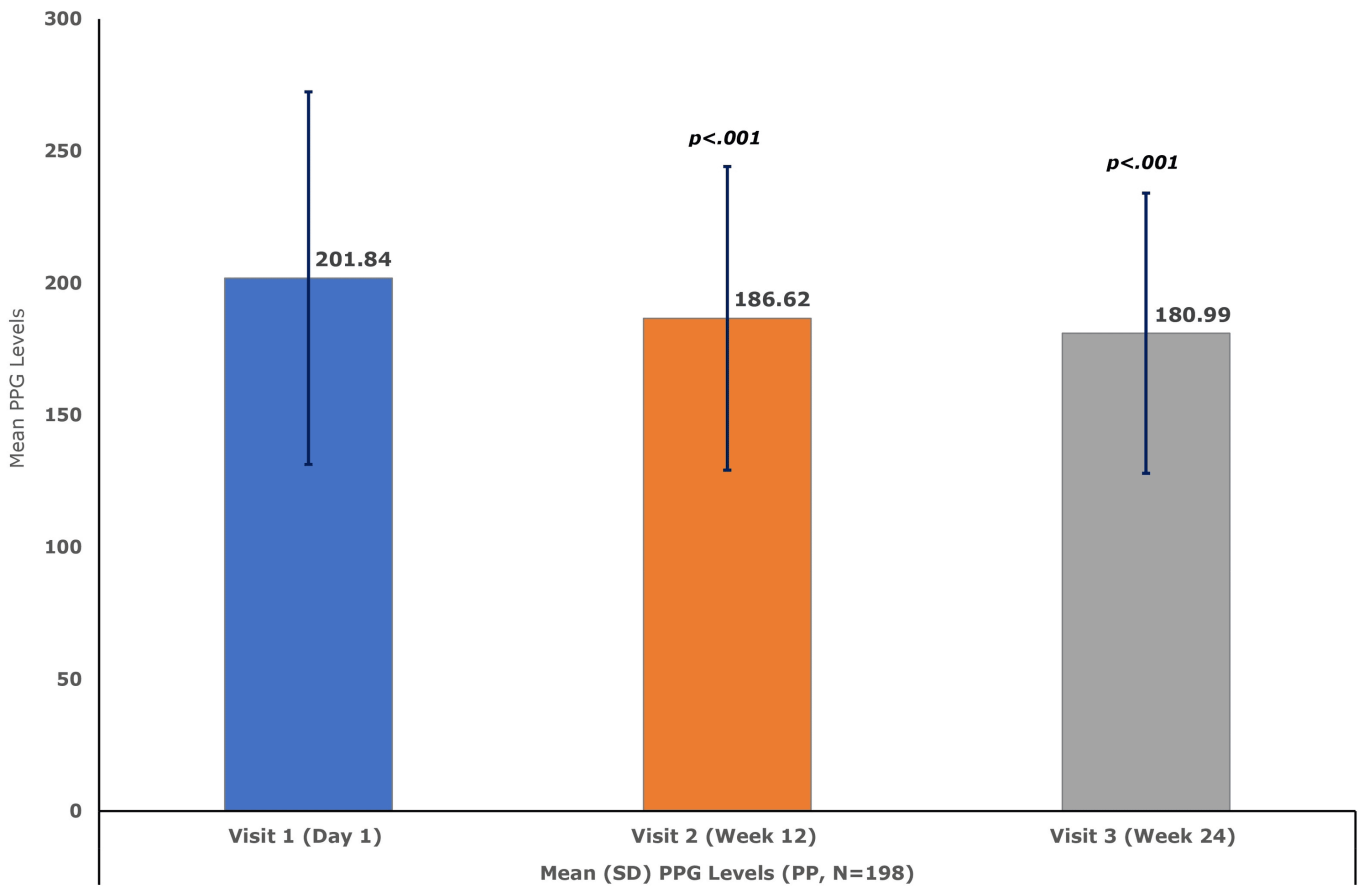
Mean (SD) postprandial glucose levels (PP set, N = 198) N is the number of patients in the PP set. PPG, postprandial glucose; PP, per protocol; SD, standard deviation.

**Figure 4:**
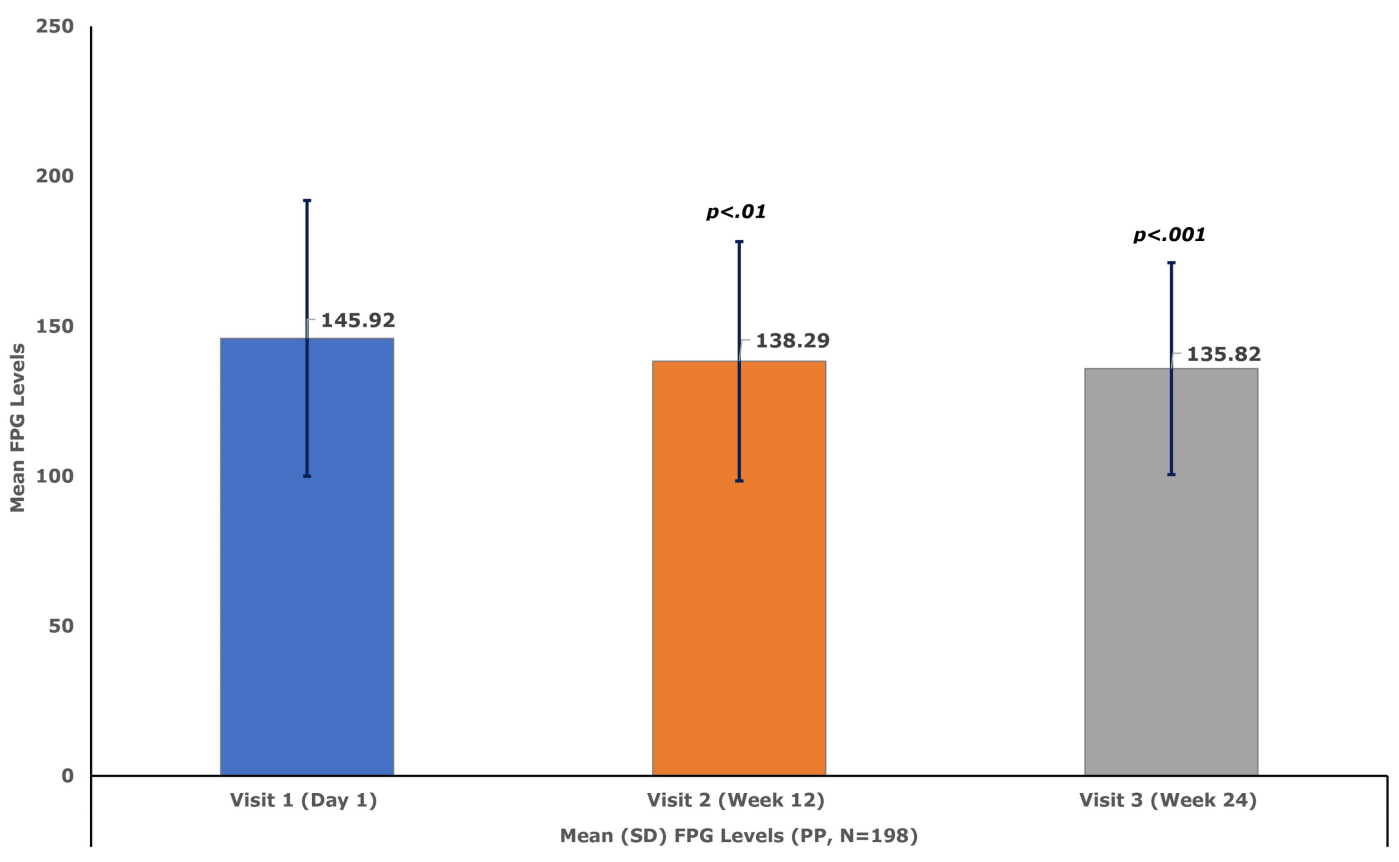
Mean (SD) fasting glucose levels (PP set, N = 198) N is the number of patients in the PP set. FPG, fasting plasma glucose; PP, per protocol; SD, standard deviation.

The mean (SD) FPG declined significantly by 7.63 (33.97) mg/dL (95% CI, −11.7 to −3.6; p<0.01) at week 12 and by 10.10 (35.97) mg/dL (95% CI, −13.93 to −6.27; p<0.001) at week 24, compared to the baseline mean (SD) level of 145.92 (45.98) mg/dL (Figure 3). Similarly, compared to the baseline mean (SD) PPG level of 201.84 (70.56) mg/dL, the PPG level decreased significantly by 15.22 (55.04) mg/dL (95% CI, −21.37 to −9.06; p<0.001) at week 12 and by 20.84 (62.31) mg/dL (95% CI, −27.20 to −14.48; p<0.001) at week 24 (Figure 4). 

The study findings demonstrate that a 24-week regimen of once-daily vildagliptin 100 mg SR was well tolerated, with hepatic and renal function parameters remaining stable, as indicated by stable ALT, AST, serum creatinine, serum amylase, and serum lipase levels throughout the treatment period. These outcomes support its overall safety profile in the study cohort.

Compared to the baseline mean (SD) AST level of 26.32 (7.14) U/L, AST increased slightly by 0.28 (5.11) U/L (p>0.05) at week 12 and by 0.13 (6.00) U/L (p>0.05) at week 24. In contrast, the mean (SD) ALT level decreased by 0.07 (6.33) U/L (p>0.05) at week 12 and declined significantly further by 1.32 (7.26) U/L (p<0.05) at week 24, compared to the baseline mean (SD) ALT level of 29.08 (9.69) U/L (Table 2).

Compared to the baseline mean (SD) serum creatinine level of 0.92 (0.22) mg/dL, serum creatinine decreased by 0.02 (0.16) mg/dL (p>0.05) at week 12, with no change observed at week 24. The mean (SD) serum amylase level increased slightly by 0.54 (28.02) U/L (p>0.05) at week 12 before declining significantly by 3.40 (13.52) U/L (p<0.05) at week 24, compared to the baseline value of 59.36 (30.45) U/L. Conversely, the mean (SD) serum lipase level declined significantly by 3.00 (16.93) U/L (p<0.05) at week 12 and by 3.68 (18.42) U/L (p<0.01) at week 24, from a baseline value of 49.06 (21.10) U/L (Table 2).

**Table 2:** Mean hepatic and renal parameters (PP set, N = 198) ^a^Analyzed using repeated-measures analysis of covariance (ANCOVA); pairwise comparisons performed using paired t-test. N is the number of patients in the PP set. ALT, alanine aminotransferase; AST, aspartate aminotransferase; CI, confidence interval; PP, per protocol; SD, standard deviation.

Variables	Visit 1 (Day 1), Mean±SD	Visit 2 (Week 12), Mean±SD	t-statistic	Mean±SD, Difference(95% CI)	P-value^a^	Visit 3 (Week 24), Mean±SD	t-statistic	Mean±SD, Difference(95% CI)	P-value^a^
AST (U/L)	26.32±7.14	26.60±5.46	0.776	0.28±5.11 (-0.27, 0.83)	>0.05	26.45±5.34	0.312	0.13±6.00 (-0.48, 0.75)	>0.05
ALT (U/L)	29.08±9.69	29.01±7.46	0.156	-0.07±6.33 (-0.76, -0.61)	>0.05	27.76±7.26	2.565	-1.32±7.26 (-2.08, -0.56)	<0.05
Serum creatinine (mg/dL)	0.92±0.22	0.90±0.17	1.622	-0.02±0.16 (-0.04, 0.00)	>0.05	0.92±0.19	0.067	0.0±0.19 (-0.02, 0.02)	>0.05
Serum amylase (U/L)	59.36±30.45	59.90±18.80	0.270	0.54±28.02 (-1.84, 2.92)	>0.05	56.50±15.99	2.282	-3.40±13.52 (-5.17, -1.63)	<0.05
Serum lipase (U/L)	49.06±21.10	46.06±14.55	2.498	-3.00±16.93 (-4.64, -1.38)	<0.05	45.38±11.56	2.815	-3.68±18.42 (-5.10, -2.27)	<0.01

## Discussion

This 24-week prospective study showed that once-daily vildagliptin 100 mg SR monotherapy was well tolerated in patients with T2DM, with no serious adverse drug reactions reported. All observed events were mild and non-serious in nature. Hepatic and renal function parameters remained stable throughout the study period. Improvements in glycemic parameters, including HbA1c, FPG, and PPG, were observed at 12 and 24 weeks. These findings support the use of vildagliptin 100 mg SR once daily as a treatment option in routine clinical practice.

The goal of SR drugs is to reduce the peak-to-trough fluctuations of drug concentrations, which allows for less frequent administration of the drug. This is achieved by decreasing the rate of drug release, thus also reducing its rate of absorption [25]. Regulations usually require comparing the bioequivalence of SR formulations with immediate-release (IR) formulations when the SR product is meant to substitute for the IR product. The goal is to show an equivalent extent of absorption (AUC) and acceptable peak exposure. Previous studies have compared the PK and PD profiles of vildagliptin 100 mg SR with vildagliptin IR 50 mg. The pharmacokinetic parameters, including peak plasma concentration (Cmax), area under the plasma concentration-time curve from 0 hours to the last measurable concentration (AUC0-t), and AUC-time curve up to infinity (AUC0-inf), were not significantly different between the two formulations [9]. Both formulations also had similar PK and PD profiles under fed conditions [2, 5] and fasting conditions [5] in healthy adults. Moreover, vildagliptin 100 mg SR demonstrated >80% DPP-4 inhibition for approximately 23 hours [3, 9]. It is usually assumed that if two formulations are bioequivalent, this also implies therapeutic equivalence [25].

However, the clinical efficacy of vildagliptin 100 mg SR monotherapy has been demonstrated only in one retrospective study. That study involved 3316 patients who were prescribed vildagliptin 100 mg SR once daily, with data collected through an electronic case report form. After a 3-month follow-up, the mean HbA1c level decreased by 0.7% (8.0% to 7.3%, p<0.001) [20]. In our study, the mean decrease in HbA1c was 0.62%, which is similar to the previous study despite a much smaller sample size. A prospective phase 4 study in 120 Indian patients compared the efficacy and safety of vildagliptin 50 mg twice daily and vildagliptin 100 mg SR OD added to metformin 1000 mg/day in two divided doses [19]. The patients in that study had a higher mean HbA1c of 9.17%. At 12 weeks, it decreased by 2.53%, which is higher than that observed in our study. However, this difference may be influenced by the higher baseline HbA1c and differences in treatment approach. The study also studied the safety profile by evaluating the hepatic and renal parameters [19], and similar to our results, no clinically meaningful changes were observed. These findings further support the favorable safety profile of vildagliptin 100 mg SR once daily.

Another study compared the effect of vildagliptin 100 mg SR OD versus sitagliptin 100 mg, with or without metformin, in Indian patients [26]. The decrease in glycemic parameters was greater with vildagliptin 100 mg SR OD. At 12 weeks, the mean HbA1c decreased by 1.35% with vildagliptin versus a 1.13% decrease with sitagliptin. The decrease in mean FPG and PPG was also greater in the vildagliptin group. That study also evaluated hepatic and renal parameters and found no significant change at 12 weeks [26], consistent with the overall safety findings observed in our study. Two other studies have also reported 12-week outcomes of vildagliptin 100 mg SR OD combined with metformin in Indian patients [17, 18]. The efficacy of the combination in improving glycemic parameters at 12 weeks with statistical significance was reported by both. However, our study is the first to report 24-week outcomes of vildagliptin 100 mg SR OD. Our patients were close to the glycemic target of HbA1c <7 at week 24 (mean HbA1c at 24 weeks was 7.06%). The ADA 2025 guidelines recommend considering early combination therapy rather than a stepwise approach for patients presenting with HbA1c levels 1.5-2% above the target. The mean HbA1c of our patients at baseline was 7.89%, indicating that they could be treated with monotherapy. Our study shows that vildagliptin 100 mg SR OD is a safe and effective treatment for patients with T2DM who are candidates for monotherapy.

This study is subject to certain limitations, primarily the absence of a comparative arm, which restricts the ability to evaluate clinical outcomes against vildagliptin 50 mg twice daily. Although the sample size was relatively small, it was determined based on statistical considerations and was considered adequate to meet the study objectives. Nonetheless, these findings represent meaningful preliminary evidence and warrant further validation through future prospective studies in larger and more diverse populations.

## Conclusions

This study demonstrated that vildagliptin 100 mg SR once-daily monotherapy administered over 24 weeks was safe and effective in patients with T2DM, providing consistent glycemic control without significant adverse events or evidence of renal or hepatic toxicity. The treatment was well tolerated, with all reported adverse drug reactions being mild and non-serious, and resulted in statistically significant improvements in key glycemic parameters, including HbA1c, fasting plasma glucose, and postprandial glucose. These findings support vildagliptin 100 mg SR as an effective, once-daily therapeutic option for patients with T2DM who are suitable candidates for monotherapy.
